# Adolescent views on participating in HIV biomedical research during pregnancy: a qualitative analysis of motivators and barriers

**DOI:** 10.1080/09540121.2026.2628310

**Published:** 2026-02-12

**Authors:** Hadas Clementine Baron, Rita Vanessa Masese, Aamirah Mussa, Annie Thom, Pearson Mmodzi, Mary Kasule, Ontibile Tshume, Raksha Rajeshmohan, Aishah Hagan-Bezgin, Picrina Winner, Mbabi Bapabi, Kehumile Ramontshonyana, Kristen Sullivan, Chelsea Morroni, Agatha Bula, Anne Drapkin Lyerly, Suzanne Day

**Affiliations:** a Department of Social Medicine, Center for Bioethics, The University of North Carolina at Chapel Hill, Chapel Hill, NC, USA; b Botswana Harvard AIDS Institute Partnership, Gaborone, Botswana; c Usher Institute, University of Edinburgh, Edinburgh, UK; d UNC Project Malawi, Lilongwe, Malawi; e Botswana-Baylor Children’s Clinical Centre of Excellence, Gaborone, Botswana; f School of Public Health, University of Michigan, Ann Arbor, USA; g Southport Hospital, Mersey & West Lancashire Teaching Hospitals NHS Trust, Liverpool, UK; h Centre for Reproductive Health, University of Edinburgh, Edinburgh, UK; i Department of Population Health and Pathobiology, Global One Health Academy, North Carolina State University, Raleigh, NC, USA; j Department of Medicine, Division of Infectious Diseases, The University of North Carolina at Chapel Hill, Chapel Hill, NC, USA

**Keywords:** HIV, pregnancy, adolescents, ethics, clinical trial participation, good health and well-being, reduced inequalities

## Abstract

Pregnant adolescents are often excluded from biomedical HIV research participation, resulting in less evidence to inform safe and effective treatment and prevention strategies. We explored adolescent views on enrollment in biomedical HIV research during pregnancy via in-depth interviews with ever-pregnant adolescents living with and at-risk of HIV age 15–20 years in Botswana and Malawi. A semi-structured interview guide explored decision-making around study enrollment during pregnancy using vignettes depicting two hypothetical HIV studies: (1) testing pregnancy-specific dosage of an HIV medication, and (2) a randomized control trial comparing an oral regimen to a new injectable. Audio-recorded interviews were transcribed, translated to English, coded in NVivo, and thematically analyzed for emergent themes in participation motivators/barriers. Of the 80 adolescents interviewed (40 living with and 40 at-risk of HIV), 73 (91.3%) were interested in joining vignette study 1 and 65 (81.3%) in vignette 2. Participation motivators included treating/preventing HIV, gaining health knowledge, and helping others. Barriers included study participation requirements, the studies’ experimental nature, and randomization. Adolescents’ high interest in and reasons for participation during pregnancy suggest their exclusion from biomedical HIV research may be inconsistent with their views, interests, and capacities, providing important considerations for ethical and inclusive study design.

## Introduction

Despite growing recognition of the urgent need to prioritize the interests and concerns of adolescents in drug trials (Faruqui et al., 2024), significant gaps remain for the inclusion of pregnant adolescents in clinical HIV research (Wickremsinhe et al., 2018). These gaps hinder the evidence base for HIV prevention and treatment strategies for adolescents during pregnancy and limit their access to optimal care (World Health Organization (WHO) 2021; Lyerly et al., 2021). This is particularly concerning given that pregnant adolescents face increased risk of HIV acquisition (Thomson et al., 2018), heightened risks of maternal and fetal mortality and morbidity (González et al., 2017), and higher rates of vertical transmission compared to adult women living with HIV (Ramraj et al., 2018). Both children and pregnant people have enhanced regulatory protections for engaging in research, though there is uncertainty regarding how such protections apply at the intersection of these populations (Knopf et al., 2020; Wickremsinhe et al., 2018). For example, US regulations governing research in pregnancy, Subpart B, does not address the age of people who are pregnant, and Subpart D, which governs research in children, does not mention pregnancy (Wickremsinhe et al., 2018). Similarly, regulatory guidance in Malawi and Botswana refer broadly to pregnant women without specifying age, thereby overlooking the unique needs and vulnerabilities of pregnant adolescents (Malawi Ministry of Health., 2011, Botswana Ministry of Health 2011; National Institute of Allergy and Infectious Diseases, 2024). This lack of specificity creates a regulatory gap for those who are at the intersection of childhood and pregnancy, potentially leaving pregnant adolescents without clear ethical or procedural guidance for their inclusion in research (Malawi Ministry of Health,, 2011; Botswana Ministry of Health 2011; Barchi & Little, 2016; National Institute of Allergy and Infectious Diseases, 2024). Pregnant people have historically been excluded from clinical trials due to concerns about potential teratogenic effects and adverse pregnancy outcomes, and adolescents who are pregnant face additional research participation hurdles (Frew, Saint-Victor, Brewinski Isaacs, et al., 2014).

Amidst these complexities, little is known about adolescents’ interest in or willingness to participate in clinical research during pregnancy. This knowledge gap is of particular concern in sub-Saharan Africa, where adolescent girls are twice as likely to be living with HIV than boys of the same age, and where HIV clinical research is increasingly conducted among adolescents (Karim & Baxter, 2019;; Murewanhema et al., 2022 Toska et al., 2020). Adolescent girls in sub-Saharan Africa also experience the highest rates of pregnancy globally, further underscoring the need for focused studies that explore their interest in and potential concerns about HIV clinical research participation (Toska et al., 2020). To address this need, we examined adolescents’ perspectives on biomedical HIV research participation during pregnancy at two sites with high rates of adolescent HIV acquisition and where HIV research is commonly conducted (Botswana and Malawi). The aim of this study was to surface adolescents’ motivations and concerns pertaining to the idea of participating in biomedical HIV research during pregnancy in order to inform the ethical inclusion of this population in HIV prevention and treatment research.

## Materials and methods

We conducted in-depth interviews with adolescents aged 15–20 years old who had experienced pregnancy at any point in their lives (including at the time of interview) and were either living with HIV or at-risk of HIV acquisition. Interviews were designed to explore decision-making around clinical HIV study enrollment during pregnancy using two vignettes depicting hypothetical HIV studies, tailored to participants’ HIV status: (1) testing the pregnancy specific dosage (pharmacokinetics, PK) of a medication to prevent/treat HIV, and (2) a randomized control trial (RCT) comparing an established oral regimen to a new injectable to treat/prevent HIV, which had not been previously studied in pregnancy. Vignettes were developed initially by the study team and then revised based on input from an expert in adolescent research engagement, who reviewed them for age-appropriateness, clarity, and simplicity (bearing in mind the need to also translate study materials) and developed visual aids to assist with understanding. Study team members based in Malawi and Botswana also provided input to ensure clarity and contextual appropriateness in both settings before implementation. Our qualitative study received ethical review and approval from the University of North Carolina at Chapel Hill Institutional Review Board (protocol #22–2256), the Health Research Development Committee of the Botswana Ministry of Health (#00976), the Botswana-Baylor Centre of Excellence Institutional Review Board (#BBCO-IRB-2210–38), and the Malawi National Health Sciences Research Committee (protocol #20/09/305).

### Participants/Setting

Individuals were eligible to participate if they were (1) aged 15–20 years, (2) currently pregnant or had ever experienced pregnancy, and (3) living with HIV or at risk of HIV acquisition. We defined “at-risk” for HIV acquisition based on having current or prior experience with pregnancy in adolescence, which is associated with STI/HIV acquisition, and living in an area identified as being one of elevated HIV risk for adolescent girls and young women (Thomson et al., 2018). There is wide variation in how adolescence is defined, with varying and dynamic legal, cultural, and scientific definitions for the age range encompassed by this period of human development (Curtis, 2015; Sawyer et al., 2018). In addition, the experiential boundaries of adolescence defy age cutoffs, as those who have only recently surpassed the age of majority can still relate to the experiences of legal minors. For these reasons, and in keeping with recent literature calling for a broader view of this developmental phase (Sawyer et al., 2018), we adopted the age range of 15–20 years to define adolescence.

In Botswana, adolescents were recruited from District Health Management Team clinics and a pediatric clinic in Gaborone using two approaches: (1) authorized clinic staff used electronic health and paper records to flag potentially eligible adolescents to approach for recruitment, and those who were eligible were invited to participate via phone call; and (2) the study team members approached potential participants in the clinic while they were attending routine clinic visits and invited them to participate. Adolescents living with HIV were also recruited from a local peer support group for adolescents living with HIV. In Malawi, potential participants were identified through electronic health records, routine clinic visits at an antenatal clinic in Lilongwe, drop-in centers, or were referred by peer leaders.

### Informed consent

Parental/guardian informed consent and adolescent assent were provided before interviews with participants who were legal minors, while adolescents of legal age provided independent informed consent. We obtained verbal informed consent from parents/guardians and adolescents who were not legal minors in Botswana and written informed consent from parents/guardians and adolescents who were not legal minors in Malawi. Adolescents who were legal minors in both settings provided verbal assent to participation.

### Data collection

Interviews were conducted in-person by experienced, trained qualitative interviewers fluent in English and the primary local language (Chichewa in Malawi, Setswana in Botswana). Interviews were conducted using a semi-structured interview guide designed to explore a variety of topics related to participation in HIV research during pregnancy. Part of the guide (and the focus of the present analysis) involved the use of two vignettes designed to explore adolescents’ initial receptiveness to and considerations in participating in a hypothetical biomedical HIV prevention or treatment study (with treatment/prevention posed as the study purpose depending on the adolescent’s HIV status). Vignette, interview guide and consent form translations from English into Chichewa and Setswana were conducted by local team members, local translation committees, and contract translators.

Vignettes were read aloud to participants and included overviews of the hypothetical studies’ designs, activities involved in participation, and both established and unknown safety data (see Supplemental Information 1). Interviewers tailored the vignette text to suit participant comprehension, and participants paused to ask questions during the vignette explanation. Participants were asked to imagine that they were 17 years old if they were 18 years old or older, and all were asked to imagine that they were pregnant for each vignette. Participants were then asked to describe their thoughts about participating in the vignette studies, with questions probing both motivators and concerns about joining. Illustrations designed by a member of the study team with expertise in youth programming were shown during the vignette explanations to facilitate understanding (see Supplemental Information 2). Participants were also shown unused vials to provide a better understanding of the medical blood draws that are involved in biomedical research. Participants could pause, ask questions of the interviewer, and seek clarification on any details of the vignette before proceeding with the interview guide questions and at any point during the interview questions. After each interview, demographic data were verbally collected from participants using a questionnaire that was completed by the interviewer. Participants received monetary compensation commensurate with local standards ($10.00 USD in Malawi and 50 Pula, or approximately $4.00 USD in Botswana) to compensate for their time and transportation.

### Data analysis

All interviews were audio-recorded, then professionally translated and transcribed verbatim from the local languages of Setswana and Chichewa to English, as needed. Transcriptions were reviewed by members of the study team to ensure accuracy. The development of the codebook was an iterative and collaborative process involving multiple team members. An initial set of topical parent codes (e.g., reasons to participate, reasons to decline) was developed a priori based on broader questions asked in the interview guide, with subsequent subcodes and refinement of codes developed inductively through close reading of the transcripts by the team to finalize the codebook. A minimum of 20% of all transcripts were team-coded, involving three coders independently coding the same transcript and then meeting to compare coding and discuss and resolve discrepancies. The remaining transcripts were then divided up and coded by one coder, with a second coder checking for fidelity; notes were made on discrepancies and resolved through consensus among the coding team. Coding was conducted using NVivo qualitative analysis software. Using a thematic analysis approach (Braun & Clarke, 2006), the coded data were analyzed to identify emergent themes in participants’ decision-making processes regarding participating in the study vignettes. This included reasons for willingness to join the hypothetical studies as well as reasons to decline participation, with emergent themes in willingness/disinterest analyzed across the two vignettes; we also identified emergent themes in willingness/disinterest that were vignette-specific.

Since the two vignettes were focused on participation in experimental trials, with variations in prevention or treatment only to ensure relevance to all interviewees, we combined data from all participants for thematic analysis to focus the analysis on adolescents’ reasons for interest/disinterest in study participation, rather than on comparing differences in participation interest by HIV status. Likewise, analyses were combined for adolescents living in Botswana and Malawi, as the goal of this exploratory analysis was to examine the views of adolescents from regions with high rates of adolescent HIV and adolescent pregnancy, rather than develop a comparative analysis. Demographic data were consolidated using Microsoft Excel spreadsheets and analyzed descriptively. The study adhered to the Consolidated Criteria for Reporting Qualitative Research (COREQ) guidelines (Tong et al., 2007).

## Results

### Participant demographics

Interviews were conducted with 80 adolescents in total; 40 in Botswana and 40 in Malawi, composed of 20 adolescents living with HIV and 20 at-risk of HIV acquisition at each site. Participant demographics are reflected in Table 1. Almost half (47.5%) were aged 15–17 years old, and 52.5% were 18–20 years old. Most participants had completed some school in grades 9–12 (76.3%). Almost all participants described themselves as Christian (92.5%) and most were single at the time of the interview (60%).

### Willingness to participate in HIV vignette studies

Most adolescents reported willingness to join at least one of the two vignette studies (Table 2). The majority of adolescents (91.3%) expressed that they would be willing to participate in vignette 1 involving testing a new medication to prevent/treat HIV that has not yet been studied in pregnant adolescents; six (7.5%) declined to participate, and one adolescent was unsure. Compared to vignette 1, slightly more adolescents (10, 12.5%) indicated they would not join vignette 2 (testing a new injection using an RCT study design), and four adolescents were unsure. However, most participants still agreed to participate in vignette 2 (66, 82.5%).

### Thematic analysis

The following themes represent adolescents’ reasoning for their decisions to either participate in or decline involvement in the studies, and are summarized as motivators and barriers below in Table 3.

### Motivators for vignette participation

Five themes were identified across vignettes that influenced motivation to participate in the vignette studies among adolescents: access to HIV prevention/management, gaining more knowledge about improving their health, contributing to science and helping others, trust in the study design, and receiving access to tangible benefits.

### Access to HIV prevention/management

Overwhelmingly, the majority of adolescents described participation in the vignette studies as a valuable opportunity to gain access to new medications that could protect the health of themselves and their baby by helping to better manage their HIV (if they were an adolescent living with HIV, LWHIV) or preventing HIV acquisition (if they were an adolescent at-risk of HIV acquisition, ARHA). As one participant explained, the idea of joining was appealing “because [she] would be preventing [her] child and [herself] from dangers of diseases that can infect [her]” (*age 17, ARHA, Botswana*). Adolescents provided various explanations for why the new medications in the vignettes would be beneficial to their health. For example, adolescents living with HIV described how an injectable option for HIV treatment would make their treatment easier to manage, easier to adhere to, and result in fewer clinic visits:

... Maybe it can benefit me in a way that sometimes I forget to take my pills, or if I sleep early I miss a pill ... So the injection can make things easier for me because I will not have the pill burden. (age 18, LWHIV, Malawi)

This one for injection is good because they write for you that come after 2 months for your injection, unlike the everyday pill. You can forget. You can go to visit people. With a pill you can make a mistake. (age 19, LWHIV Botswana)

Many adolescents at risk of HIV also expressed that study participation would result in access to HIV prevention medication that would provide protection from HIV acquisition. For example, when asked why she would participate, this participant shared:

I can [come] to get the medication ... it can help me ... so that the baby can grow with health ... my body should be protected from HIV as well as my baby. (age 17, ARHA, Malawi)

One adolescent further explained that the study would be bringing “modern things” *(age 17, LWHIV, Malawi)* in the form of new medications, representing an improvement or advancement in HIV care.

Many adolescents also noted the perceived risk of HIV exposure to the fetus during pregnancy, explaining that access to HIV prevention or management medication via study participation would not only be beneficial to their own health, but would also “[help] the baby to not be born with the virus I have” *(age 20, LWHIV, Botswana)*.

Because if I join, it is going to help so that the child I will be pregnant with cannot be infected with HIV. (age 16, LWHIV, Botswana)

Mainly it is protection on the unborn baby. It’s a painful thing to contract HIV from parents ... This brings the problem of explaining to a child that he or she was born with HIV because he or she wasn’t protected ... you give the child the medication but what will happen if the friends notice the medication. So mainly we should protect the unborn children because by doing so we will protect the next generation. (age 18, ARHA, Malawi)

While many adolescents expressed motivation to join specifically to access new HIV prevention or treatment medication, some participants shared that their motivation was rooted in the more general reason of wanting to protect the health of themselves and their babies. For these adolescents, trial participation was linked with better health outcomes overall, not only in relation to HIV. For example, one participant shared that they would want to join the study “because I would want ... to take care of my health and the infant” *(age 17, ARHA, Botswana)*.

### Gaining health knowledge

While HIV prevention and management were major considerations in vignette study participation decision-making, adolescents also commonly expressed a motivation to gain more knowledge about their own health and bodies. One such form of health knowledge was understanding how their bodies process the new medication, so they would know the proper medication to take. Others also expressed that by participating in the vignettes, they would understand the effect of the trial medication on both themselves and their fetuses. For example, two participants expressed motivation to join the study because they would be better prepared for their current or future pregnancies.

For those who don’t know how it works may be concerned on how it works, that maybe it can cause harm to the baby, but for me I will not be concerned since I will have the knowledge of its benefits to me and the baby. (age 18, ARHA, Malawi)

For some participants, participating in research was an opportunity to learn about pregnancy health and the prevention of HIV for themselves and their fetus.

When I am pregnant, I have to know ... which method to protect me and my child so that our health can be a good one. If I am pregnant and I just sit and not join this program, I am going to stay at home without knowledge and I don’t have any skill to protect me and my child from HIV. (age 20, ARHA, Botswana)

### Contributing to science and helping others

Helping others by contributing to the science of HIV research was another prominent motivator. Adolescents particularly noted that the study results would provide much-needed data on medication safety, helping other pregnant adolescents to manage or prevent HIV. As one adolescent described, through her participation in the vignette study, much could be learned that would help others in the future:

Yes, I would want to take part that they should test the medicine on my body, that if they find that the medicine has worked on me and I will not get HIV, it will make the other people that would come after me to benefit from the medicine. And it would also make other pregnant women to be given these medications, since it will be like they are testing it on me so that they see how the medicine is working or what challenges would they be. (age 17, ARHA, Malawi)

Some participants also shared that while there may not be a direct nor immediate benefit for themselves at the time of participation, it could benefit people in the future – “not just [for] women who are not pregnant” *(age 19, LWHIV, Botswana*), but also others who are living with HIV:

[Taking a daily tablet] is a job in young people in general ... Injection 6 times a year is something, we will not feel it, we will not complain ... maybe in the future they will, they will, yes, leave the tablets and continue with the injection. (age 19, LWHIV, Botswana)

Another adolescent noted that her participation would contribute to the benefit of all Malawians and future generations of her nation, in addition to those who are close to her.

What motivates me is that I am Malawian and this is going to help Malawians who are here today and the babies we are expecting to be born. So I can take part because it might help me, my relatives and anyone close. (age 19, ARHA, Malawi)

Adolescents also felt their participation could help others directly by enabling adolescents to share the knowledge they would gain through study participation. They described how participation could help them encourage their peers to participate in HIV-related research during pregnancy. They could also directly share new knowledge gained from study participation about taking medication to treat/prevent HIV in pregnancy. As one adolescent stated, “the time that my pregnant friends do not know [about the medication], I would explain it to them” *(age 18, ARHA, Malawi)*. By participating in the vignette study, they could “encourage [their] peers from what [they have] learned here” (*age 19, LWHIV, Malawi*), to the direct benefit of others in their lives and communities.

### Trust in the study design

Trust in the study design also emerged as a motivation for adolescents’ willingness to join the vignette studies. One aspect of trust was expressed through a belief that the vignette studies posed a low risk of potential harm to study participants. For example, a participant supported her decision to participate by explaining that her pregnancy would not be affected by the low dose of medication administered in the study, with doses being so low “that it cannot be passed to the baby during pregnancy” (age 19, LWHIV, Malawi). Some participants also noted their trust that risks would be mitigated, or that if any concerns arose during study participation, they could stop taking the medication or receive help if needed. For these participants, the ability to pause or change medications with medical supervision during study participation was reassuring:

So, it’s up to you to tell the person who is giving you the medicine if the medicine is not working well. You can tell the doctor the problems you are facing and the doctor can change your medicine. (age 17, LWHIV, Malawi)

Other aspects of trust related to adolescents’ perceptions of the study design. When asked what was important to them when deciding to join the vignette study, several participants expressed that they specifically liked how the study was designed to include a high degree of care for participants, and that caution would be taken in monitoring the medication in their bodies. For example, an adolescent described how ongoing monitoring during study participation would be reassuring to her:

It is because they are protecting me by extracting my blood and seeing how the medicine is working in my body. So, they are protecting me and the unborn baby so that there shouldn’t be any mistake. (age 17, LWHIV, Malawi)

Similarly, another participant expressed appreciation for the umbilical cord blood sampling technique, highlighting it as a valuable approach to the study design that would allow for close monitoring of fetal health:

Drawing blood from the umbilical cord to check the dosage of medication from mother to the child, I think it is a very good idea. (age 19, LWHIV, Botswana)

### Receiving access to additional tangible benefits from trial participation

Some participants also highlighted tangible benefits that would be accessible if they participated in the study. These benefits included enhanced health monitoring and counseling and reimbursement for transportation. One participant specifically shared that she would want to join the study to access “things like counselling, when [she] needs counselling” (*age 19, LWHIV, Botswana*). Some described the advantage of being closely monitored in the trial as a reason for joining; for example, a participant noted that additional health issues may be caught in the process of monitoring throughout study participation via routine blood collection:

Once blood is drawn you can also find out other medical complications that the baby has. (age 16, ARHA, Malawi)

### Barriers to vignette participation

Participants weighed perceived benefits and risks of study participation, and while most participants were motivated to join both vignette studies, they also identified important barriers. Barriers to participation across the two vignettes included experimental uncertainties, and aversion to study activities. An additional barrier that emerged specific to vignette 2 (an RCT comparing an established oral regimen to a new injectable to treat/prevent HIV not previously studied in pregnant adolescents) was concerns with the randomization process.

### Experimental uncertainties

A prominent theme in barriers mentioned was hesitancy over experimental uncertainties. Several participants were concerned about the lack of safety data in participating in the two vignette studies. For example, one adolescent who declined participation noted that her decision was “mainly [made] because of fear” of the unknowns associated with an experimental medication *(age 18, ARHA, Malawi)*.

The problem comes where you have two drugs, on one hand you have pills which they already know that they work and on the other hand an injection which they don’t know. With the injection you can’t be certain of the outcomes. This is the main challenge. (age 19, ARHA Malawi)

Participants also cited uncertainties about the medication’s effectiveness in preventing or treating HIV, with consequences for participants, especially HIV transmission to the fetus should the medicine prove ineffective (specifically for adolescents living with HIV). One participant noted that “having a baby that is born infected is what scares me the most” *(age 17, ARHA, Malawi)*.

Some also expressed concerns about the impact of changing their medications to participate in the vignette studies, without knowing if “there might be a problem or not” *(age 18, LWHIV, Malawi)* regarding how their body might react. Finally, some raised concerns about whether the experimental medication might impact their pregnancy, including the potential for birth defects or pregnancy loss.

I can just have that fear that they want to test how the medication can work on me and the baby so it can be difficult for me to accept because maybe it can cause harm to me or maybe the baby can born with deformities. (age 18, ARHA, Malawi)

### Physical and logistical burdens

Several participants also noted that certain study activities would discourage their participation, including blood draws, forms of medication administration, and logistical demands. For example, some participants were hesitant about the volume of blood to be collected in vignette study 1, which they perceived to be harmful to their health:

For instance, when they are taking samples for viral load, its mostly one vial and in this case four is a lot of bottles. (age 15, LWHIV, Malawi)

Some expressed concerns regarding the method of medication administration with a strong preference for one method over the other, which would preclude their participation in a study in which they could not take their preferred method of medication. For example, some participants preferred pills over injections due to a fear of needles, while others, in contrast, preferred the ease of long-acting injections over daily pills.

I am afraid of an injection that is why I was talking about the tablet. (age 17, ARHA, Botswana)

The pills, the issue of taking it daily is tiresome. A lot of us people we tend to forget and some are also reluctant to take medication. (age 18, ARHA, Malawi)

Logistical demands included travel coordination, with a few adolescents noting it would be troublesome to “have to go back to the hospital many times to get your blood collected” (*age 17, LWHIV, Botswana*).

### Randomization process

A concern that emerged exclusively in relation to vignette 2 was the randomization process for RCT participation. Several adolescents were concerned about not being able to decide about which drug they would receive, with the removal of choice seen as increasing the risks of participation. For example, one participant noted that randomization increases risk by assigning trial participants to a study group in which the administered medication might not be best suited to the individual, resulting in the risk of physical harm should the assigned medication provoke an adverse response:

... It is literally putting a patient’s life at risk. What if, what if a patient is to do that like, the computer decides for them which medication to take, but your body does not respond well to the medication. It kind of puts the patient in an uncomfortable spot or a risky spot. (age 17, LWHIV, Botswana)

Other participants expressed a desire to know which medication they would take before agreeing to participate, which is not possible under randomized study conditions. As one adolescent explained when asked why she had declined interest in participating in vignette 2, “you would not know which side you will be at ... it will be through randomization” *(age 18, AHIVA, Malawi)*.

## Discussion

This qualitative study aimed to surface adolescent perspectives on participating in biomedical HIV research during pregnancy. We found high levels of willingness to join two vignette studies among our adolescent participants; most adolescents indicated they would join at least one study during pregnancy, with vignette 1 (new medication testing) being the most acceptable study. Though these findings are promising for recruitment into HIV biomedical studies, further exploration of our key findings in relation to the specific context of adolescence, and pregnancy in adolescence, suggests how clinical HIV research could better advance ethical inclusion of this population.

Many participants expressed a deep trust in research, recounting a belief that the vignette studies were well-designed and safe to join (despite our explanation of unknowns and experimental conditions), and that close medical supervision would provide protection from study harms. This expression of trust contrasts with literature on participation in research during pregnancy among adults (DiClemente et al., 2010) as well as on adolescent HIV research participation (Frew, Saint-Victor, Isaacs, et al., 2014) which has noted that mistrust of research may pose a barrier to involvement in clinical HIV studies. This strong theme of trust suggests a belief among adolescents that adults involved in research would prioritize their well-being and safety, specifically as study participants who are pregnant. Ensuring that this trust is both warranted and upheld is thus an ethical imperative for fostering future participation in this population, as trust is closely linked with high levels of research engagement (Bailey et al., 2014; Henderson et al., 2018; Henderson et al., 2022).

Many adolescents also described a strong desire to contribute to science and help others, a motivator commonly seen in adult populations, including pregnant adults, where altruism is often a key factor in clinical research participation (MacDonald et al., 2023; Stanford et al., 2002). However, a facet unique to adolescents was a strong desire to share knowledge with their peers, motivated by the opportunity to help immediate others with the knowledge learned from study participation. This suggests that motivation for study participation among adolescents may be rooted more in peer relationships than in solidarity with other pregnant people, which contrasts with findings from previous studies with pregnant adults (Sullivan et al., 2020). Relationship-building, identity formation, and connection with peers are defining features of adolescent development (Ragelienė, 2016). To leverage this altruistic motivator, studies should be designed to engage adolescents as key players in the research process, making them active contributors of the study team (Azzopardi et al., 2024; Hawke et al., 2018). This can be facilitated by creating youth friendly research environments and offering opportunities for training and capacity building for them to take on advisory and leadership roles, such as peer facilitators, allowing young people to be valuable members of the study team (Azzopardi et al., 2024; Hawke et al., 2018). While recruiting adolescent populations is often characterized as a challenge (Azzopardi et al., 2024), our findings suggest that creating opportunities for adolescents to connect with their peers as part of the research process may facilitate their successful involvement.

Many adolescents also described gaining knowledge from research participation as a strong motivator. While others have similarly described knowledge as a motivator for adolescent research participation (Stanford et al., 2002), this may be a particularly significant facilitator during pregnancy. Adolescent pregnancy is often highly stigmatized (Hall et al., 2018; Miller et al., 2021), and this may be further compounded by the stigma of living with HIV (Kabunga et al., 2024; Oluma et al., 2025). As a result, pregnant adolescents are often socially isolated, hiding their pregnancies until term in fear of experiencing stigma (Miller et al., 2021). At the intersection of pregnancy, HIV, and adolescence, this population may experience multiple layers of stigma, which can limit their access to critical healthcare information (Hall et al., 2018; Miller et al., 2021). That the opportunity to gain health knowledge was a motivator of vignette participation among adolescents in our study may reflect a lack of access to health education, with research participation offering a valuable (and perhaps rare) opportunity to access relevant information in a welcoming environment. A study in which researchers are intentionally seeking their involvement may seem particularly welcoming, as our vignettes described studies that were specifically designed to focus on pregnant adolescents as the study population. Accessing crucial health information that empowers them to make informed decisions may also provide an opportunity to take more control of their own health and the health of their fetuses and future children (Oluma et al., 2025).

Despite high willingness to participate due to these motivating factors, adolescents also raised concerns about randomization and unknown risks to the fetus as barriers to vignette participation. These findings are consistent with previous research which has shown that adolescents ranked medication side effects and “being experimented on” as among the biggest barriers to study participation (Shah et al., 2020). Despite these concerns, and amidst thorough youth-friendly explanation of the study processes and unknowns, an overwhelming majority of adolescents still expressed interest in participation. These findings reinforce that many adolescents have the capacity to weigh the benefits and risks of medical interventions and experimental research conditions. They also highlight areas in which youth-friendly and pregnancy-specific information about study risks might be particularly important (Alexander et al., 2015). Our findings further suggest that rote exclusion of pregnant adolescents should be reconsidered as being incongruent with adolescents’ willingness and capacity to participate, supporting efforts that have been made to address the exclusion of pregnant adolescents in HIV biomedical research (Bekker et al., 2024; DiClemente et al., 2010; Knopf et al., 2020; MacDonald et al., 2023; Stanford et al., 2002; Wickremsinhe et al., 2018). One recent example of such efforts is the PURPOSE 1 trial, in which cisgender girls and women who became pregnant were allowed to remain enrolled in the study (Bekker et al., 2024). Regulatory bodies and future biomedical research should consider ways in which to leverage the motivators and address the barriers identified by adolescents so their ethical inclusion in research is supported and sustained.

Our findings also highlight barriers that are readily addressable to improve adolescent inclusion in research. While adolescents did raise concerns about the experimental conditions of the vignettes and potential risks associated with new medications, many of their concerns were more general, relating to common clinical research requirements (e.g., blood draws). We further found that for some adolescents, logistical barriers, rather than fear of experimental risks, may be the primary barrier to participation in clinical research. To address these barriers and increase adolescent inclusion, study designs should be made more responsive to the concerns and needs of adolescents, conducted in youth-friendly settings. Engaging adolescents in the planning, design, and execution of research may result in study designs that are optimized to account for adolescents’ lived experiences (Azzopardi et al., 2024; Hawke et al., 2018). Given the unique social and cultural contexts of pregnancy in adolescence (Frew, Saint-Victor, Brewinski Isaacs, et al., 2014; Karim & Baxter, 2019;; Murewanhema et al., 2022 Ramraj et al., 2018), it is of further importance to involve adolescents with experience of pregnancy in informing study design.

There are four main limitations in our study. First, participation decisions in hypothetical study vignettes may not necessarily reflect real-life decisions about whether to participate. However, the vignettes were designed to reflect in detail the characteristics of clinical studies that adolescents could potentially join to better understand the kinds of factors that influence adolescent decision-making. Thus, while willingness to participate may differ in real life, our use of vignettes is still able to capture adolescents’ perspectives on the motivators and barriers of study participation that would be relevant to them in considering whether to participate. Second, our sample is limited to adolescents in Botswana and Malawi and thus is not necessarily generalizable to other geographic or cultural contexts. However, these are two sites of high interest for addressing the HIV epidemic in youth and conducting clinical HIV research. Third, our recruitment methods may have resulted in a sample of adolescents who are highly trusting of medical staff and clinical research and thus biased towards high willingness to participate in clinical studies. This likewise indicates that our study captured the views of adolescents who would be most likely to be among those voluntarily recruited for study participation, thus providing insights into how the concerns, needs, and priorities of those most likely to participate in future clinical research could be addressed in order to foster greater inclusion of adolescents in future research. Finally, while participants were asked to share their individual perspectives, and while interviewers presented participants with accessible details to help add realistic considerations to the study vignettes, adolescents’ participation decisions may not fully reflect the complexities of real-life circumstances. For example, adolescents may not have sole decision-making authority in real life, depending on regulatory and legal factors and familial dynamics. As such, our findings may not fully capture the constraints that exist in research settings. Our findings further offer important perspectives to consider amidst growing awareness of the disproportionate impact of HIV on adolescents who experience pregnancy in eastern and southern Africa (Groves et al., 2022).

Adolescent participants identified key motivators and concerns that impact their willingness to participate in biomedical HIV research during pregnancy. Understanding the views of this population is important to improving their inclusion in research and addressing the underrepresentation of this population in HIV treatment and prevention data. The adolescents we interviewed were both motivated to participate and thoughtful in their rationales and consideration of risks in expressing their desire whether to do so. Our findings regarding adolescents’ concerns suggest several important avenues for future research that would help to leverage the motivators identified and mitigate their concerns with research participation. These include investigating the best ways to communicate about risk mitigation strategies in biomedical research with adolescent participants, with specific consideration of adolescents’ concerns related to participation during pregnancy. Additionally, further research is needed investigating how best to develop biomedical research protocols and settings that would be more supportive of adolescent participants in general, and pregnant adolescent participants specifically (e.g., how best to create peer connection opportunities for study participants, and how to mitigate logistical barriers to pregnant adolescent participation). In combination with the present findings, such studies would provide important data for developing study protocols and research guidance that would support the ethical and responsible inclusion of pregnant adolescents in HIV treatment and prevention research.

## Supplementary Material

Supp 1

Supp 2

## Figures and Tables

**Table 1.** Demographics of adolescent interview participants (N = 80).

Demographic Characteristics	n	(%)

**Age, mean (SD)**	17.8	(1.2)
**Age, years**		
15–17	38	(47.5)
18–20	42	(52.5)
**Location**		
Malawi	40	(50)
Botswana	40	(50)
**Highest education level completed**		
Grades 1–8	18	(22.5)
Grades 9–12	61	(76.3)
Post-Secondary	0	(0)
None	1	(1.25)
**Religion**		
Christianity	74	(92.5)
Islam	3	(3.75)
Other	4	(5)
**Relationship status**		
Single	48	(60)
Partner, not living together	21	(26.25)
Partner, living together	10	(12.5)
Married	1	(1.25)

**Table 2.** Overall willingness to participate in two hypothetical HIV related studies among adolescents interviewed (N = 80).

Vignette Participation Willingness	Prevention Version (ARHA) n (%)	Treatment Version (LWHIV) n (%)	Total n (%)

**Vignette 1. New medication**			
Would participate	36 (45%)	37 (46.3%)	73 (91.3%)
Would not participate	4 (5%)	2 (2.5%)	6 (7.5%)
Unsure/it depends	0	1 (1.3%)	1 (1.3%)
Data missing	0	0	0
**Vignette 2. RCT: daily pills vs. injectable**			
Would participate	31 (38.8%)	34, (42.5%)	65 (81.3%)
Would not participate	6 (7.5%)	4, (5%)	10 (12.5%)
Unsure/it depends	3 (3.8%)	1, (1.3%)	4 (5%)
Data missing	0	1, (1.3%)	1 (1.3%)

**Table 3.** Summary of the thematic analysis of motivators and barriers to participation in the vignette studies.

Thematic Category	Response Themes

Motivators of study participation	• Access to HIV prevention/management • Gaining health knowledge • Contributing to science/helping others • Trust in study design • Receiving tangible participation benefits
Barriers to study participation	• Experimental uncertainties • Burdens of study requirements • Randomization process
