# Supplementary material for: Adolescent views on participating in HIV biomedical research during pregnancy: a qualitative analysis of motivators and barriers
Source: AIDS Care. Author manuscript; Available in PMC 2026 Mar 27. (PMC13025434; doi:10.1080/09540121.2026.2628310)
Supplement: Supp 1 [file NIHMS2150128-supplement-Supp_1.docx]

**Supplemental Information 1. Text used by interviewers to explain study vignettes for participants living with HIV and at-risk of HIV (original English version).**

| 1. New medication – for those living with HIV [Vignette 1]   *Here is the first study to imagine.* [***Imagine you are 17 years old.]*** *Imagine you are pregnant and you are taking a medicine to treat your HIV. You get asked whether you would be willing to join a study that is trying to get information about a new medication to treat HIV which is not normally used in pregnant women but which might be in the future. The new medication has been tested in teenagers and women who are not pregnant and has worked well and been safe for them. A small number of women have gotten pregnant while taking this medication, but there are still a lot of questions about how it will work in pregnancy. The goal of the study is to figure out how much of this medicine should be given to pregnant women in the future so that it will be enough, but not too much, to safely treat the HIV and protect the baby.*  *If you join the study, you can continue your current medication and take a small dose of the new medication twice during pregnancy and another dose several weeks after your baby is born. The new medication would be at such a small dose that it is very unlikely there would any harm or benefit to you to taking it. The benefit of the research would be for other people (and maybe for you, if you get pregnant again) in the future. The study would involve your coming to the clinic to have your blood drawn to test how your body is processing the new medicine.*  *On the days that you take the new medicine, you would come to clinic for most of the day. You would have a small tube of your blood collected four times, twice in the morning, and twice in the afternoon. The small amount of blood to be collected is safe for you and the baby [****SHOW VIAL]****. When you give birth, the doctors will collect some blood from the umbilical cord that connects you and your baby. This is done to test how much of the medicine is going from the mother to the baby. You and your baby will not feel any pain when the blood is collected from the umbilical cord, and it will not hurt you or your baby in any way. A tube of blood will also be collected from your baby when he/she is one week old at the same time as the one-week check-up clinic visit. It will not cost you anything to be part of in this study, and your transportation costs for study visits will be covered.*   1. New medication – for those at risk of HIV [Vignette 1]   *Here is the first study to imagine. [Imagine you are 17 years old.] Imagine you are pregnant. You get asked whether you would be willing to join a study that is trying to get information about a new medication to prevent HIV which is not normally used in pregnant women but which might be in the future. The new medication has been tested in teenagers and women who are not pregnant and has worked well and been safe for them. A small number of women have gotten pregnant while taking this medication, but there are still a lot of questions about how it will work in pregnancy. The goal of the study is to figure out how much of this medicine should be given to pregnant women in the future so that it will be enough, but not too much, to safely prevent her from getting HIV and also protect the baby.*  *IF PARTICIPANT IS CURRENTLY TAKING PREP SAY: “You can continue your current medication.”*  *CONTINUE FOR ALL:*  *If you join the study, you will take a small dose of the new medication twice during pregnancy and another dose several weeks after your baby is born. The new medication would be at such a small dose that it is very unlikely there would any harm or benefit to you to taking it. The benefit of the research would be for other people (and maybe for you, if you get pregnant again) in the future. The study would involve your coming to the clinic to have your blood drawn to test how your body is processing the new medicine.*  *On the days that you take the new medicine, you would come to clinic for most of the day. You would have a small tube of your blood collected four times, twice in the morning, and twice in the afternoon. The small amount of blood to be collected is safe for you and the baby [SHOW VIAL]. When you give birth, the doctors will collect some blood from the umbilical cord that connects you and your baby. This is done to test how much of the medicine is going from the mother to the baby. You and your baby will not feel any pain when the blood is collected from the umbilical cord, and it will not hurt you or your baby in any way. A tube of blood will also be collected from your baby when he/she is one week old at the same time as the one-week check-up clinic visit. It will not cost you anything to be part of in this study, and your transportation costs for study visits will be covered.* |
| --- |
| 1. RCT testing a new injection – for those living with HIV [Vignette 2]   *Here is the last study to imagine.* [***Imagine you are 17 years old.]*** *Imagine you are pregnant and you are taking a medicine to treat the HIV. You get asked whether you would be willing to join a study that is testing whether the medicine you are currently taking is better or worse than a new medicine to treat HIV. The new medicine is an injection of an anti-HIV drug that only has to be given every two months. People living with HIV getting the new injectable medicine no longer take daily pills. The injectable HIV medication has been tested in teenagers and women who are not pregnant and has worked well and been safe for them. A small number of women have gotten pregnant while taking this injectable medication and they and their babies did not have any problems, but there are still a lot of questions about how it will work in pregnancy and if it is safe. The new medicine may have different side effects, no-one knows yet. Your doctor doesn't know whether you will do better on your current medicine or on the new one. You might do better on the new drug or have fewer side effects, but you might not.*  *This study is looking at two groups – one group keeps taking their current medicine and the other group has injections. If you join the study, a computer will decide which group you are in - this is called randomization – you do not have a say. So you might continue taking your daily medicine or you might have the injection every 2 months This means the researchers can easily compare different medicine and see which one works better. It’s like putting your hand into a bag with two balls in it. If you pull out the red ball, you stay on your current medicine, and if you pull out the blue ball, you get the other medicine. You don’t know whether you will get the red ball or the blue ball, but you know you will get a ball and therefore some kind of medicine. Like before, it will not cost you anything to be part of in this study, and your transportation costs for study visits will be covered.*   1. RCT testing a new injection – for those at risk of HIV [Vignette 2]   *Here is the last study to imagine. [Imagine you are 17 years old.] You are pregnant. You get asked whether you would join a study looking at two groups - one will take a daily anti-HIV pill and the other will have an injectable anti-HIV drug every two months. The daily anti-HIV pill has been shown to work and be safe for pregnant women. The injectable anti-HIV medication has been tested in teenagers and women who are not pregnant and has worked well and been safe for them. A small number of women have gotten pregnant while taking this injectable medication and they and their babies did not have any problems, but there are still a lot of questions about how it will work in pregnancy and if it is safe.*  *This study is looking at two groups – one group takes the daily pill and the other group has injections. If you join the study, a computer will decide which group you are in - this is called randomization – you do not have a say. So you might take the daily tablet or have the injection every 2 months. This means the researchers can easily compare different medicine and see which one works better. It’s like putting your hand, without looking, into a bag with two balls in it. If you pull out the red ball, you get one medicine, and if you pull out the blue ball, you get the other medicine. You don’t know whether you will get the red ball or the blue ball, but you know you will get a ball and therefore some kind of medicine. Like before, it will not cost you anything to be part of in this study, and your transportation costs for study visits will be covered.* |
