# Supplementary material for: Adolescent views on participating in HIV biomedical research during pregnancy: a qualitative analysis of motivators and barriers
Source: AIDS Care. Author manuscript; Available in PMC 2026 Mar 27. (PMC13025434; doi:10.1080/09540121.2026.2628310)

**Supplemental Information 2. Illustrations used in interviews to explain vignette studies (original English version).**


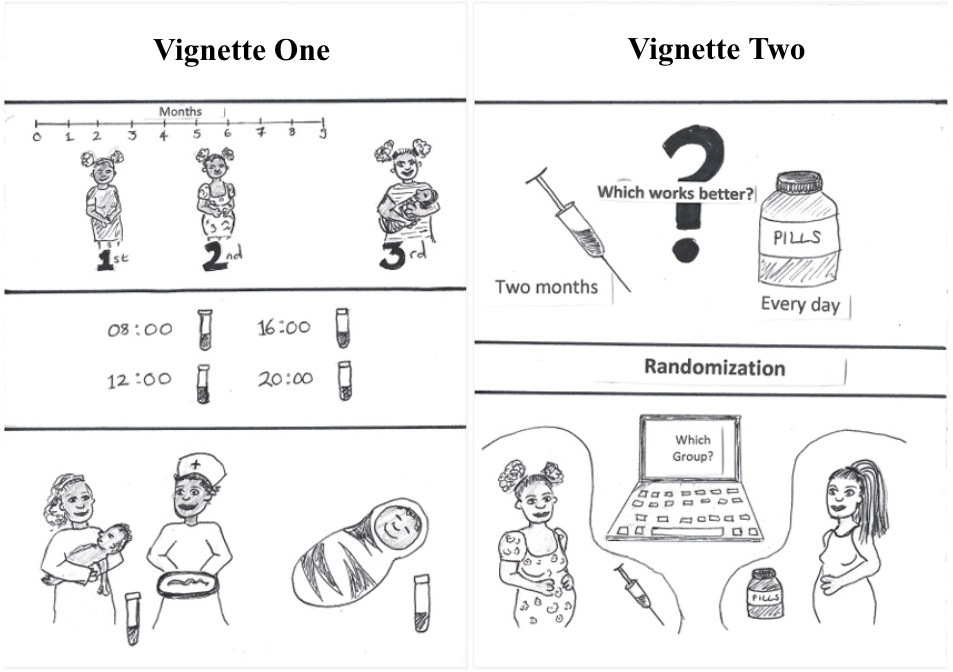

Supplement: Supp 2 [file NIHMS2150128-supplement-Supp_2.docx]
